# Serum Tumor Necrosis Factor-Alpha Levels in Acute Leukemia and Its Prognostic Significance

**DOI:** 10.7759/cureus.24835

**Published:** 2022-05-08

**Authors:** Sapana Verma, Anurag Singh, Geeta Yadav, Rashmi Kushwaha, Wahid Ali, Shailendra P Verma, U.S. Singh

**Affiliations:** 1 Department of Pathology, King George's Medical University, Lucknow, IND; 2 Department of Hematology and Oncology/Clinical Hematology, King George's Medical University, Lucknow, IND

**Keywords:** tumor necrosis factor-alpha, flow cytometry, elisa, bone marrow examination, leukemia

## Abstract

Introduction

Tumor necrosis factor-alpha (TNF-α) is a pleiotropic cytokine that facilitates malignant cells in immune evasion, survival, and treatment resistance by generating a favorable milieu for them. It is shown to be ectopically produced by malignant/leukemic and immune cells in the tumor microenvironment, providing a tumor-supportive environment and playing an important part in the establishment and progression of malignant cells. It is linked to hyperleukocytosis, high blast count, and poor clinical outcomes in acute leukemia (AL). Considering the varied role and different expression patterns of tumor necrosis factor-alpha in acute leukemia and its clinical relevance, the present study was planned to monitor the level of tumor necrosis factor-alpha in patients with acute leukemia and its correlation with disease outcome. The aim of this study was to monitor the level of tumor necrosis factor-alpha in patients with acute leukemia at the time of diagnosis and after induction chemotherapy.

Material and methods

The study included cases classified as acute leukemia based on morphological examination, bone marrow analysis, and flow cytometry. In all patients with acute leukemia (n = 90) and controls (n = 10), the serum tumor necrosis factor-alpha level was measured using a Diaclone Human ELISA kit (Diaclone, Besancon, France) (solid phase sandwich ELISA) at diagnosis and after induction chemotherapy.

Results

Tumor necrosis factor-alpha levels were substantially higher in T-acute lymphoblastic leukemia (T-ALL) cases, followed by acute myeloid leukemia (AML) and B-acute lymphoblastic leukemia (B-ALL), at the time of diagnosis, compared to the control. A significant reduction in serum tumor necrosis factor-alpha level was seen in patients with acute leukemia after induction phase chemotherapy (P < 0.05). Tumor necrosis factor-alpha levels were considerably reduced (P < 0.001) in the majority of acute leukemia cases after the induction phase, while high tumor necrosis factor-alpha levels were positively correlated with incomplete remission status in the remaining cases.

Conclusion

Tumor necrosis factor-alpha is involved in the progression of acute leukemia and its relapse. High levels of tumor necrosis factor-alpha are linked to leukocytosis, high blast counts, and worse survival in patients with acute leukemia. Monitoring of tumor necrosis factor-alpha may be helpful in patients with acute leukemia in view of available antitumor necrosis factor-alpha therapy.

## Introduction

Tumor necrosis factor-alpha (TNF-α) is a cytokine with a wide range of functions. It is engaged in all stages of leukemogenesis, including cellular proliferation, transformation, angiogenesis, inflammation, and extramedullary infiltration, and plays a crucial role in the development and progression of malignant illness. TNF-α is also critical in the creation of a tumor microenvironment and aids malignant cells in immune evasion, survival, and treatment resistance [1]. TNF-α is activated by two receptors: TNF-α receptor type-1 (TNFR-1), also known as P55 or P60, and TNF-α receptor type-2 (TNFR-2), also known as P75 or P80 [2]. TNFR-1 is expressed by a variety of cell types, but TNFR-2 is expressed only by immune and endothelial cells [3,4]. Activated macrophages and immune cells such as T-lymphocytes, natural killer cells, and neutrophils release TNF-α. TNF-α is shown to be ectopically produced by malignant/leukemic and immune cells in the tumor microenvironment, providing a tumor-supportive atmosphere and playing a significant role in the establishment and progression of malignant disease [5]. TNF-α is associated with poor clinical outcomes in acute leukemia (AL). TNF-α is related to early hyperleukocytosis in acute myeloid leukemia (AML) and acute lymphoblastic leukemia (ALL) [4,6,7]. TNF-α expression levels were linked to a greater percentage of blasts in AML, extramedullary infiltration, and an unfavorable prognosis in acute leukemia [6]. Other researchers found that patients with AML with lower levels of TNF-α had a greater rate of complete remission and event-free survival. However, TNF-α levels were not found to be an independent predictor of clinical outcomes [8]. TNF-α promotes leukemia cell survival and development by activating the nuclear factor kappa B (NF-KB) and c-Jun N-terminal kinase/activator protein-1 (JNK/AP-1) pathways, which inhibit apoptosis and promote proliferation [9,10]. TNF-α promotes tumor cell survival and treatment resistance in AML by upregulating heme oxygenase-1 (HO-1) without activating nuclear factor kappa B (NF-KB) [11]. In melanoma, TNF-α signaling via the TNFR-1 receptor causes activated CD8+ T-cells to die and suppresses T-cell-mediated tumor rejection [12]. TNFR-2 receptor signaling mediates pro-tumor activities, which in turn mediates Treg proliferation in tumors [13,14]. TNFR-1 and TNFR-2 signaling appear to be critical for controlling tumor-promoting actions by generating a favorable tumor microenvironment. TNF-α has been shown to serve a dual role in cancer research. Because of TNF-α's diverse role and expression pattern in AL, as well as its clinical importance, the current study was designed to measure the level of TNF-α in patients with AL at the time of diagnosis and after chemotherapy and its correlation with disease outcome.

## Materials and methods

Study setting

This was a prospective study conducted at the Department of Pathology, in collaboration with the Department of Clinical Hematology and Department of Pediatrics, King George's Medical University, Lucknow, Uttar Pradesh. The study duration was of one year between June 2019 and July 2020. The study was approved by the institutional ethics committee (651/Ethics/19) on May 21, 2019.

Inclusion and exclusion criteria

The study included cases diagnosed as AL based on morphological examination, bone marrow analysis, and flow cytometry and those who opted for chemotherapy. Only those patients who agreed to participate in the study were enrolled. Written informed consent was taken from all patients. Patients who refused to give their consent or who chose not to receive chemotherapy were excluded from the study. Patients with malignancies other than AML and ALL, as well as those on chemotherapeutic drugs, were also excluded from the study.

Study design

The study was a prospective observational study.

Data collection

Case sheets of patients were used to record a detailed clinical history and examination findings. A complete blood count was performed for blast percentage, and a peripheral blood smear (PBS) was examined for blast morphology. Patients and/or close relatives gave written informed consent for bone marrow examination (BME). All patients had BME, which included both bone marrow aspiration (BMA) and bone marrow biopsy (BMB), from the posterior superior iliac spine under strict aseptic conditions using a standard protocol. Leishman stain was used to stain BMA smears. For blast immunophenotyping, 0.5 mL bone marrow aspirate was collected and processed as per the stain-lyse-wash protocol. Acquisition and analysis were done on Becton Dickinson (BD) FACS Canto II Flow Cytometer (Becton Dickinson, San Jose, CA, USA) using the FACS Diva software. Cases were categorized as AML, B-acute lymphoblastic leukemia (B-ALL), or T-acute lymphoblastic leukemia (T-ALL) based on immunophenotyping. In all instances with AL and controls, the serum TNF-α level was measured using a Diaclone Human ELISA kit (Diaclone, Besancon, France) (solid phase sandwich ELISA) according to the manufacturer's instructions.

Statistical analysis

Statistical analysis was done using the Statistical Package for Social Sciences (SPSS) software version 21.0 (IBM Corp., Armonk, NY, USA), and data were presented as percentages and means ± standard deviations (SDs). The ANOVA test was used to compare the within-group and between-group variances among the study groups. Post hoc analysis was done for each mean comparison. P-value < 0.05 was taken as significant.

## Results

The study included 90 cases of acute leukemia and 10 age- and sex-matched normal controls. On the basis of immunophenotyping of 90 cases, 32 cases were diagnosed as AML, 53 as B-ALL, and five as T-ALL. The mean age of cases was 17.85 ± 17.07 years (range: 1-74 years). The mean age of B-ALL (11.83 ± 13.62 years) and T-ALL (12.80 ± 7.86 years) cases were found to be significantly lower than that of AML cases (26.11 ± 20.21 years) (P = 0.005). The male/female ratio of cases was 2:1, and there was no significant difference noted between different groups.

Hematological parameters and TNF-α level at the time of diagnosis

There was a significant difference in the hemoglobin (P < 0.001) and platelet counts (P < 0.001) of controls when compared to distinct leukemic subgroups of AML, B-ALL, and T-ALL. At the time of diagnosis, TNF-α levels were significantly raised in T-ALL cases, followed by AML and B-ALL, as compared to the control group. Differences in the TNF-α levels of controls and different subgroups of acute leukemia cases were found to be statistically significant (P = 0.038) (Table 1).

**Table 1:** Hematological parameters and TNF-α level in AML, B-ALL, and T-ALL cases at the time of diagnosis

Parameters	Control (n = 10) (mean ± SD)	AML (n = 32) (mean ± SD)	B-ALL (n = 53) (mean ± SD)	T-ALL (n = 5) (mean ± SD)	P-value
Hemoglobin (g/dL)	12.94 ± 0.69	6.91 ± 1.90	6.62 ± 2.08	7.10 ± 2.29	<0.001
Total leukocyte count (×10^9^/L)	6.64 ± 0.88	41.25 ± 83.25	43.76 ± 63.40	49.46 ± 57.22	0.442
Platelet count (×10^9^/L)	253.00 ± 65.84	70.91 ± 81.48	41.67 ± 53.37	31.40 ± 15.65	<0.001
% of blasts in PBS	NA	42.69 ± 36.12	48.25 ± 34.68	64.00 ± 32.76	0.427
% of blasts in BM	NA	68.31 ± 28.21	82.68 ± 19.78	91.00 ± 5.79	0.010
Serum TNF-α (ng/L)	16.72 ± 6.83	149.20 ± 156.64	134.82 ± 189.56	301.06 ± 345.37	0.038

Hematological parameters and TNF-α level after induction phase chemotherapy

After induction phase chemotherapy, different subgroups of acute leukemia cases showed significant differences in hemoglobin (P < 0.001) and platelet counts (P < 0.001) as compared to controls. For the rest of the parameters, change was not found to be statistically significant (Table 2).

**Table 2:** Hematological parameters and TNF-α level in AML, B-ALL, and T-ALL cases after induction chemotherapy

Parameters	Control (n = 10) (mean ± SD)	AML (n = 32) (mean ± SD)	B-ALL (n = 53) (mean ± SD)	T-ALL (n = 5) (mean ± SD)	P-value
Hemoglobin (g/dL)	12.94 ± 0.69	7.25 ± 1.10	7.33 ± 0.94	8.04 ± 1.09	<0.001
Total leukocyte count (×10^9^/L)	6.64 ± 0.88	5.98 ± 11.08	5.63 ± 7.88	10.10 ± 14.93	0.766
Platelet count (×10^9^/L)	253.00 ± 65.84	71.72 ± 38.13	54.89 ± 31.53	63.00 ± 19.24	<0.001
% of blasts in PBS	NA	1.91 ± 5.45	2.23 ± 5.83	0.00 ± 0.00	0.690
% of blasts in BM	NA	7.16 ± 8.96	10.62 ± 12.37	1.40 ± 1.67	0.116
Serum TNF-α (ng/L)	16.72 ± 6.83	90.52 ± 109.52	90.68 ± 135.99	121.24 ± 202.21	0.316

After induction phase chemotherapy in AML cases, there was a significant reduction noted for total leucocyte count (TLC) (P = 0.010) and blast percentage (P < 0.001). A significant decrease in serum TNF-α level was also observed after induction chemotherapy (P = 0.009). There was an improvement in hemoglobin in acute leukemia cases post-induction chemotherapy, and the difference was statistically significant. In the B-ALL subgroup, after induction phase chemotherapy, there was a significant increase observed in hemoglobin (P = 0.003) and platelet count (P = 0.001). A significant reduction was also noted for TLC (P < 0.001) and percentage blasts (P < 0.001). There was a significant decrease in serum TNF-α level observed after induction chemotherapy (P = 0.042). After induction phase chemotherapy in the T-ALL subgroup, there was a significant increase noted in platelet count (P = 0.023). A significant reduction was also noted for percentage blast. The reduction in serum TNF-α level was 59.73%, which was not found to be statistically significant (P = 0.212); this may be due to the small sample size of this subgroup. In the rest of the parameters, change was not found to be statistically significant (Table 3).

**Table 3:** Hematological parameters and TNF-α level in AML, B-ALL, and T-ALL cases

Parameters	AML cases (n = 32)			B-ALL cases (n = 53)			T-ALL cases (n = 5)		
	Pre-chemotherapy (mean ± SD)	Pre-chemotherapy (mean ± SD)	P-value	Pre-chemotherapy (mean ± SD)	Pre-chemotherapy (mean ± SD)	P-value	Pre-chemotherapy (mean ± SD)	Pre-chemotherapy (mean ± SD)	P-value
Hemoglobin (g/dL)	6.91 ± 1.90	7.25 ± 1.10	0.138	6.62 ± 2.08	7.33 ± 0.94	0.003	7.10 ± 2.29	8.04 ± 1.09	0.334
Total leukocyte count (×10^9^/L)	41.25 ± 83.25	5.98 ± 11.08	0.010	43.76 ± 63.40	5.63 ± 7.88	<0.001	49.46 ± 57.22	10.10 ± 14.93	0.108
Platelet count (×10^9^/L)	70.91 ± 81.48	71.72 ± 38.13	0.935	41.67 ± 53.37	54.89 ± 31.53	0.001	31.40 ± 15.65	63.00 ± 19.24	0.023
% of blasts in PBS	42.69 ± 36.12	1.91 ± 5.45	<0.001	48.25 ± 34.68	2.23 ± 5.83	<0.001	64.00 ± 32.76	0.00	0.012
% of blasts in BM	68.31 ± 28.21	7.16 ± 8.96	<0.001	82.68 ± 19.78	10.62 ± 12.37	<0.001	91.00 ± 5.79	1.40 ± 1.67	<0.001
Serum TNF-α (ng/L)	149.20 ± 156.64	90.52 ± 109.52	0.009	134.82 ± 189.56	90.68 ± 135.99	0.042	301.06 ± 345.37	121.24 ± 202.21	0.212

Correlation of TNF-α level with post-induction remission status

Of the 90 cases, complete remission was observed in 57 (63.3%). Complete remission was observed in a higher proportion of T-ALL (100%) cases as compared to AML (68.8%) and B-ALL (56.6%), although this difference was not found to be significant statistically (P = 0.115). The majority of the post-chemotherapy acute leukemia cases with lower TNF-a levels had complete remission (92.9%), while the majority of the post-chemotherapy acute leukemia cases with higher TNF-a levels had incomplete remission (85.3%). This association was found to be statistically significant (P < 0.001) (Table 4).

**Table 4:** Correlation of TNF-α level with post-induction remission status in all cases

Remission	Total (N = 90)	Post-chemotherapy high TNF-α levels (n = 34)		Post-chemotherapy low TNF-α levels (n = 56)	
		Number	%	Number	%
Remission	57	5	14.7	52	92.9
Incomplete remission	33	29	85.3	4	7.1

The majority of AML cases with post-chemotherapy lower TNF-a levels had complete remission (95.2%), while the majority of cases with post-chemotherapy higher TNF-a levels had incomplete remission (81.8%). This association was found to be statistically significant (P < 0.001). The majority of B-ALL cases with post-chemotherapy lower TNF-a levels had complete remission (90.3%), while the majority of cases with post-chemotherapy higher TNF-a levels had incomplete remission (90.9%). This association was found to be statistically significant (P < 0.001). All the cases of T-ALL had complete remission. Of the patients, 80% had post-chemotherapy lower TNF-a levels.

## Discussion

In the present study, a total of 100 subjects (10 controls and 90 cases of AML, B-ALL, and T-ALL) were included. The current study found that acute leukemia cases (all subgroups of AML, B-ALL, and T-ALL) had considerably higher TNF-α levels than healthy controls. TNF-α levels were also found to be significantly higher in patients with acute leukemia (AML, T-ALL, and B-ALL) in a study conducted by Zhou et al. [6]. It suggests that TNF-α has biological activity in patients with AL. The mean level of TNF-α was substantially greater in patients with AML than in healthy volunteers (P = 0.003) according to Sanchez-Correa et al. [15]. Potapnev et al. found that TNF-α levels in the plasma correspond with clinical characteristics and outcomes in patients with ALL. They discovered that mean TNF-α levels in patients with ALL were not substantially different from healthy children (P = 0.24) in their study. In comparison to patients with plasma levels of TNF-α below the median value, they revealed that patients with plasma levels of TNF-α above the median value had considerably greater white blood cell and blast counts in peripheral blood. As a result, their findings show that an elevated TNF-α plasma level is a useful marker for assessing disease activity/progression, but not for predicting the prognosis of childhood ALL [16]. Drabko et al. discovered that serum TNF-α levels were higher in children with ALL compared to the control group [17]. TNF-α levels were also significantly higher in ALL cases compared to the control group, and TNF-α levels following induction chemotherapy were significantly lower in patients with ALL (P = 0.05) according to Ahmed et al. [18]. In the present study, after induction chemotherapy, levels of TNF-α were significantly reduced in different leukemic subgroups but still elevated when compared to the control group. The present study showed that high levels of TNF-α positively correlated with high blast counts in AML, B-ALL, and T-ALL. Similar observations were also made by Ahmed et al. and Potapnev et al. [16,18]. Percentage blast counts significantly reduced with decreased levels of TNF-α after induction in different leukemic subgroups. High levels of TNF-α also positively correlated with percentage blast counts and high TLC. This was in concordance with the study done by Zhou et al. They observed that increased TNF-α levels were linked to higher TLC counts, extramedullary infiltration, and a poor risk group [6]. Increased serum TNF-α levels in acute leukemia cases may be due to higher blast counts according to the studies of Sanchez-Correa et al. and Potapnev et al. They discovered that TNF-α can be produced by leukemia cells, including acute myeloid leukemia (AML) [15] and acute lymphoblastic leukemia (ALL) [16], in addition to being produced by a wide variety of immune cells. Patients with lower serum TNF-α levels had lower blast percentage and lower TLC, according to the current study, and the difference between the two was statistically significant (P < 0.05). The correlation between TNF-α, blast percentage, and TLC has been taken only in a few major studies previously. In this study, TNF-α levels were seen to be higher in chemotherapy-naïve AL cases, and the levels of TNF-α significantly decreased in post-chemotherapy cases, suggesting a positive correlation of TNF-α in patients with AL corresponding to disease activity. We also noted that the majority of post-induction chemotherapy AL (AML, B-ALL, and T-ALL) cases with decreased serum TNF-α levels had complete remission. Tsimberidou et al. found that patients with lower levels of TNF-α had a higher rate of complete remission and event-free survival [8]. In our study, 85.30% of patients with AL who shared incomplete remission had high TNF-α levels (P < 0.001). TNF-α levels were found to be higher in cases of incomplete remission; however, TNF-α levels alone cannot predict illness outcomes.

There are certain limitations to our study. The sample size in the T-ALL group was very small (n = 5); hence, the statistical results cannot be true representatives of the group. Few patients have a mild increase in TNF-α level after induction chemotherapy, indicating multifactorial causes such as infection and inflammation that may lead to an increase in TNF-α level and not solely due to disease burden.

## Conclusions

TNF-α contributes to tumorigenesis by creating a tumor-supportive microenvironment. Different signaling mechanism is associated with the progression of acute leukemia and the relapse of the disease. In the present study, we found that serum TNF-α levels were significantly higher in acute leukemia cases than healthy controls at the time of diagnosis. After the induction phase, TNF-α levels were significantly decreased in the majority of acute leukemia cases, and in the rest of the cases, high levels of TNF-α positively correlated with incomplete remission status. In the present study, we conclude that a high level of TNF-α is associated with higher TLC, increased percentage of blasts, and incomplete remission in patients with acute leukemia. Accordingly, serum TNF-α levels may potentially be of clinical significance in the future, in view of follow-up and the role of anti-TNF-α therapy in acute leukemia.
